# Comprehensive analysis of differentially expressed microRNAs and mRNAs in dorsal root ganglia from streptozotocin-induced diabetic rats

**DOI:** 10.1371/journal.pone.0202696

**Published:** 2018-08-17

**Authors:** Guojun Guo, Yutian Liu, Sen Ren, Yu Kang, Dominik Duscher, Hans-Günther Machens, Zhenbing Chen

**Affiliations:** 1 Department of Hand Surgery, Union Hospital, Tongji Medical College, Huazhong University of Science and Technology, Wuhan, China; 2 Department of Plastic and Hand Surgery, Technical University of Munich, Munich, Germany; Kunming University of Science and Technology, CHINA

## Abstract

Diabetic peripheral neuropathy is a common complication associated with diabetes mellitus with a pathogenesis that is incompletely understood. By regulating RNA silencing and post-transcriptional gene expression, microRNAs participate in various biological processes and human diseases. However, the relationship between microRNAs and the progress of diabetic peripheral neuropathy still lacks a thorough exploration. Here we used microarray microRNA and mRNA expression profiling to analyze the microRNAs and mRNAs which are aberrantly expressed in dorsal root ganglia from streptozotocin-induced diabetic rats. We found that 37 microRNAs and 1357 mRNAs were differentially expressed in comparison to non-diabetic samples. Bioinformatics analysis indicated that 399 gene ontology terms and 29 Kyoto Encyclopedia of Genes and Genomes pathways were significantly enriched in diabetic rats. Additionally, a microRNA-gene network evaluation identified rno-miR-330-5p, rno-miR-17-1-3p and rno-miR-346 as important players for network regulation. Finally, quantitative real-time polymerase chain reaction analysis was used to confirm the microarray results. In conclusion, this study provides a systematic perspective of microRNA and mRNA expression in dorsal root ganglia from diabetic rats, and suggests that dysregulated microRNAs and mRNAs may be important promotors of peripheral neuropathy. Our results may be the underlying framework of future studies regarding the effect of the aberrantly expressed genes on the pathophysiology of diabetic peripheral neuropathy.

## Introduction

Diabetes mellitus (DM) is one of the most common chronic diseases around the world. Neuropathy, which is the most common chronic and debilitating complication of DM, affects approximately 50% patients with DM, usually causing pain, paresthesia, decreased mobility or even amputation [1]. The pathogenesis of diabetic peripheral neuropathy (DPN) is driven by a complex interplay of factors, such as microvascular damage and metabolic disorders. However, the exact pathophysiological processes and underlying molecular mechanisms still remain obscure [2, 3]. The streptozotocin (STZ)-induced diabetic rat is one of the most widely used diabetic animal models. Diabetic rats share numerous features identical to that of DPN patients, such as neurophysiological, functional and structural changes, and are considered a feasible animal model for studying DPN [4, 5].

MicroRNAs (miRNAs) are a cohort of small non-coding RNAs that contain approximately 22 nucleotides. They negatively regulate protein gene expression post-transcriptionally by binding to complementary sites within the 3’ untranslated region (UTR) of target mRNAs [6]. At least one conserved miRNA-binding site exist in over 60% human protein-coding genes. Taking plenty of non-conserved sites into consideration, it is believed that miRNAs control most protein-coding genes [7]. Therefore, by regulating their target genes, miRNAs are known to be associated with a wide range of human diseases, such as cancer, metabolic diseases and cardiovascular diseases [8]. Specifically, miRNAs play a crucial role in the development and progression of DM and its complications [9]. However, miRNA expression patterns and their pathological effects in DPN still lack systematic evaluation in the literature.

The dorsal root ganglion (DRG) is highly susceptible to diabetic conditions and its response to hyperglycemia leads to diabetic neuropathy [10]. There are numerous studies evaluating gene expression changes in DRGs and their roles in mediating diabetic neuropathy [11, 12], indicating the importance of DRGs in the development of DPN.

In the present study, the main objective was to identify differentially expressed miRNAs and mRNAs in diabetic DRGs, and their relationship with the pathophysiological processes of DPN. We collected DRG tissues from three pairs of STZ-induced diabetic rats and non-diabetic Sprague-Dawley (SD) rats, to conduct mRNA and miRNA expression profiling. Bioinformatics analyses, including Gene Ontology (GO) analysis, Kyoto Encyclopedia of Genes and Genomes (KEGG) pathway analysis, and miRNA-gene-network analysis, were performed to identify the significant functions and signaling pathways of the differentially expressed genes, as well as the key genes in the regulatory network.

## Materials and methods

### Animals

Twenty healthy male SD rats (180–220 g) were purchased from Experimental Animal Center, Tongji Medical College, Huazhong University of Science and Technology, Wuhan, China. The study was approved by the Animal Ethics Committee of Huazhong University of Science and Technology, and was carried out on the basis of the National Institute of Health Guidelines and Regulations. Rats were raised in separated cages where commercial rat feed and water were available ad libitum, and the room condition was kept at 23±1°C temperature, 40% humidity, and 12 hours day and night cycle as previously described [13]. The cage bedding was changed at least once a day according to the wetness of the cage bedding. The body weight was measured once a week, and the food and water intakes were measured every 2 weeks. Rats were allowed to adapt the surroundings for 1 week before the experiment was started.

### Induction of diabetes mellitus

**Fig 1:**
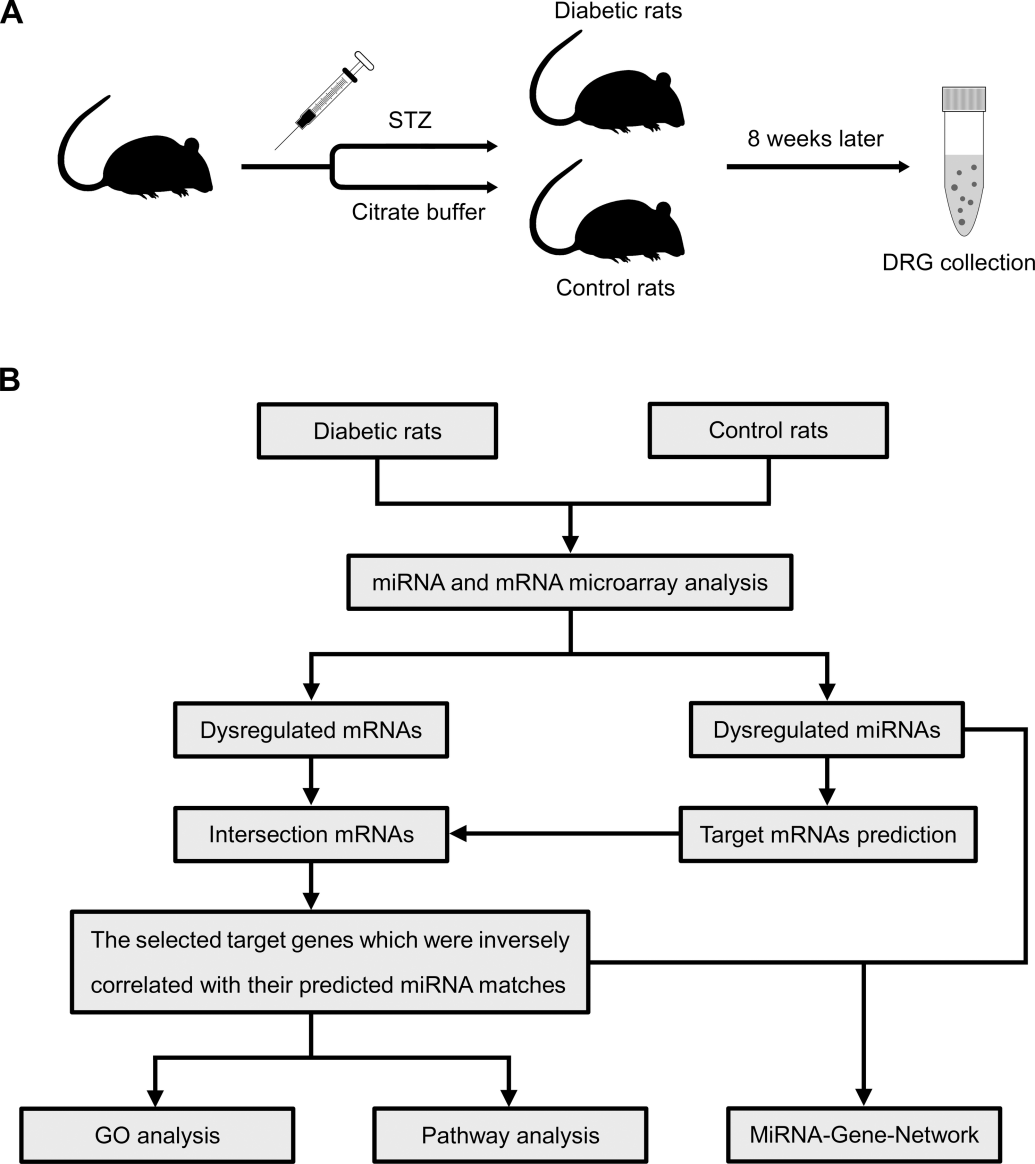
Schematic of the procedure of diabetic induction and microarray-based expression analysis. (A) Schematic overview of the procedure of diabetic rat induction and sample collections; (B) A flow chart of miRNA and mRNA microarray analysis applied in this study.

The SD rats were randomly divided into diabetic and control groups (n = 10 in each group) (Fig 1A). After a 12-hour fast, the diabetic group rats were induced by a single intraperitoneal injection of STZ (Sigma-Aldrich, St. Louis, MO, USA) at a dose of 65 mg/kg body weight, and STZ was dissolved in citrate buffer (0.1 M, pH 4.4 at 4°C) immediately before injection [13]. The control rats received an injection of an equal volume of vehicle (citrate buffer) alone. Seven days after STZ injection, nonfasting blood glucose levels were measured on tail vein blood to confirm diabetic status with a glucometer (ACCU-CHEK^®^ Active, Roche, Mannheim, Germany). The diabetic group rats with glucose levels less than 16.7 mM were excluded from this study. Rats were kept for 8 weeks following STZ injection to allow for DPN development and the glucose levels were measured once every four weeks. During this period, the diabetic rats which had significant body weight loss (cachexia) would also be excluded from this study. All excluded rats would be sacrificed using sodium pentobarbital anesthesia.

### Assessment of diabetic peripheral neuropathy

Mechanical allodynia is one of the common used behavioral indicators of DPN [14], and was assessed by testing the withdrawal threshold of the hind paws using an Electronic Von Frey device (cat. no. 38450; Ugo Basile, Gemonio, Italy) [15]. Briefly, rats were placed individually in plexiglass boxes equipped with a wire mesh floor to acclimate to the testing environment for 10 min, or enough time to cease exploratory behavior. Next, the device with a metal tip was applied through the mesh spaces to approach the plantar surface of the hind paws. The positive responses to the mechanical stimulation included sudden withdrawal, shaking, and licking of the stimulated paw, voluntary movements associated with locomotion were excluded. Both hind paws of each rat were tested, and each hind paw was tested 5 times with a 1 min interstimulus interval. The withdrawal threshold of one rat was calculated by the average force applied to both hind paws. Measurements were taken 8 weeks after STZ induction.

### RNA isolation

After the behavioral test was completed, the animals were killed by cervical dislocation and decapitation under sodium pentobarbital anesthesia. For each rat, bilateral L3-L6 DRGs were collected and pooled together as one single sample, which was immediately submerged in RNAlater^TM^ Stabilization Solution (Thermo Fisher Scientific, Vilnius, Lithuania). Each sample was stored at 4°C overnight, followed at -80°C until RNA extraction. Total RNA was extracted from DRG tissues using miRNeasy mini kit (Qiagen, Dusseldorf, Germany) in compliance with the manufacturer's protocol. RNA quality and concentration were determined by NanoDrop ND-1000 spectrophotometer (Thermo Fisher Scientific, Waltham, MA, USA), and RNA integrity was assessed by formaldehyde-agarose gel electrophoresis.

### miRNA and mRNA microarray experiments

Three diabetic rats and three control rats were chosen randomly for microarray analysis. To identify the differentially expressed miRNAs and mRNAs in diabetic and control rat DRGs, the Affymetrix GeneChip miRNA 4.0 Array and Affymetrix GeneChip Rat Transcriptome Array 1.0 were employed, respectively. The miRNA and mRNA microarray experiments and bioinformatics analysis were conducted by Genminix Informatics Co. Ltd. in Shanghai, China. The original microarray data are available at NCBI Gene Expression Omnibus with GEO Series accession number GSE110234.

### Strategy

As shown in Fig 1B, an approach with several steps was applied to analyze the dysregulated miRNA and mRNA in diabetic rats. Briefly, the random variance model (RVM) *t*-test was used to find out the differentially expressed miRNAs and mRNAs, which could raise degrees of freedom in small sample sizes [16], our filtering criteria were *P*-value<0.05 and fold-change>1.2. Then, we utilized the miRanda database to predict the potential target genes of the differentially expressed miRNAs. The intersection genes between differentially expressed mRNAs and predicted target genes were chosen. And then we collected those which were inversely correlated with their predicted miRNA matches, called the selected target genes. They were used for the following bioinformatics analyses.

### Bioinformatics analyses

Several bioinformatics analyses were performed including GO analysis, pathway analysis and miRNA-gene-network analysis. GO analysis was used to analyze the main biological function of the selected target genes. The GO database (http://www.geneontology.org) is a useful tool to organize the selected target genes into hierarchical categories (GO terms), and uncover their molecular mechanisms and biological functions [17]. A two-sided Fisher's exact test was applied to identify the significance of GO terms with *P*-value<0.05. The enrichment score of the GO terms was calculated as previously described [18]. Similarly, according to KEGG database (http://www.genome.jp/kegg/), we identified the significant pathways of the selected target genes. A Fisher's exact test was also used with *P*-value<0.05.

Additionally we combined the differentially expressed miRNAs and the selected target genes, to build the miRNA-gene-network. The relationship between the differentially expressed miRNAs and the selected target genes were evaluated according to their differential expression values, and their predicted interactions based on the Sanger miRNA database (http://www.mirbase.org/). The degree of connectivity determines the number of mRNAs regulated by a designated miRNA or the number of miRNAs which regulate a given mRNA. The larger degree of connectivity that a given miRNA or mRNA has, the more important role it plays in the regulatory network [19].

### Quantitative real-time PCR analysis

The experiment was conducted using CFX Connect Real-Time PCR detection system (Bio-Rad, USA) according to the manufacturer’s instructions on an accompanying software (CFX Manager Software). For miRNA, The M-MLV Reverse TranscriptaseiTaq^TM^ (cat. no. M170A; Promega, USA) was used for reverse transcription; for mRNA, cDNA synthesis was conducted using the iScript cDNA Synthesis Kit (cat. no. 1708890; Bio-Rad, USA). The iTaq^TM^ universal SYBR Green Supermix (cat. no. 1725124; Bio-Rad, USA) was used for quantitative detection. The miRNA and mRNA specific primers were chemically synthesized by Tianyi Huiyuan Bioscience and Technology Corporation (Beijing, China) and were listed in S1 Table. The relative expression of miRNA and mRNA were determined by U6 and GAPDH expression levels, respectively, and were calculated using the 2^-ΔΔCT^ method. All reactions were conducted in triplicate.

### Statistical analysis

The continuous variables were presented as mean±standard error (SE). To compare between two groups, the student’s t-test was used. A *P*-value<0.05 was considered statistically significant.

## Results

### Abnormalities in rats injected with STZ

One rat from diabetic group was excluded from this study because the glucose level did not meet our criterion, the rest were used in the present study. After 8 weeks following STZ injection, the nonfasting blood glucose levels, body weight and withdrawal threshold were measured to re-confirm the DPN rats were successfully induced. The nonfasting blood glucose levels of diabetic rats were greater (27.7±0.9 mM, n = 9, *P*<0.05) compared to control rats (5.8±0.2 mM; n = 10). The weight of diabetic rats was lower (241±11 g, n = 9, *P*<0.05) than that of control rats (497±8 g, n = 10). The withdrawal threshold of diabetic rats had decreased (13.8±0.6 g, n = 9, *P*<0.05) relative to that of control rats (26.4±0.2 g, n = 10), indicating mechanical allodynia. Besides, the diabetic rats also had polyphagia, polydipsia, and polyuria (based on the wet cage beddings).

### The miRNA and mRNA expression profiles in DRG tissues of diabetic rats

**Fig 2:**
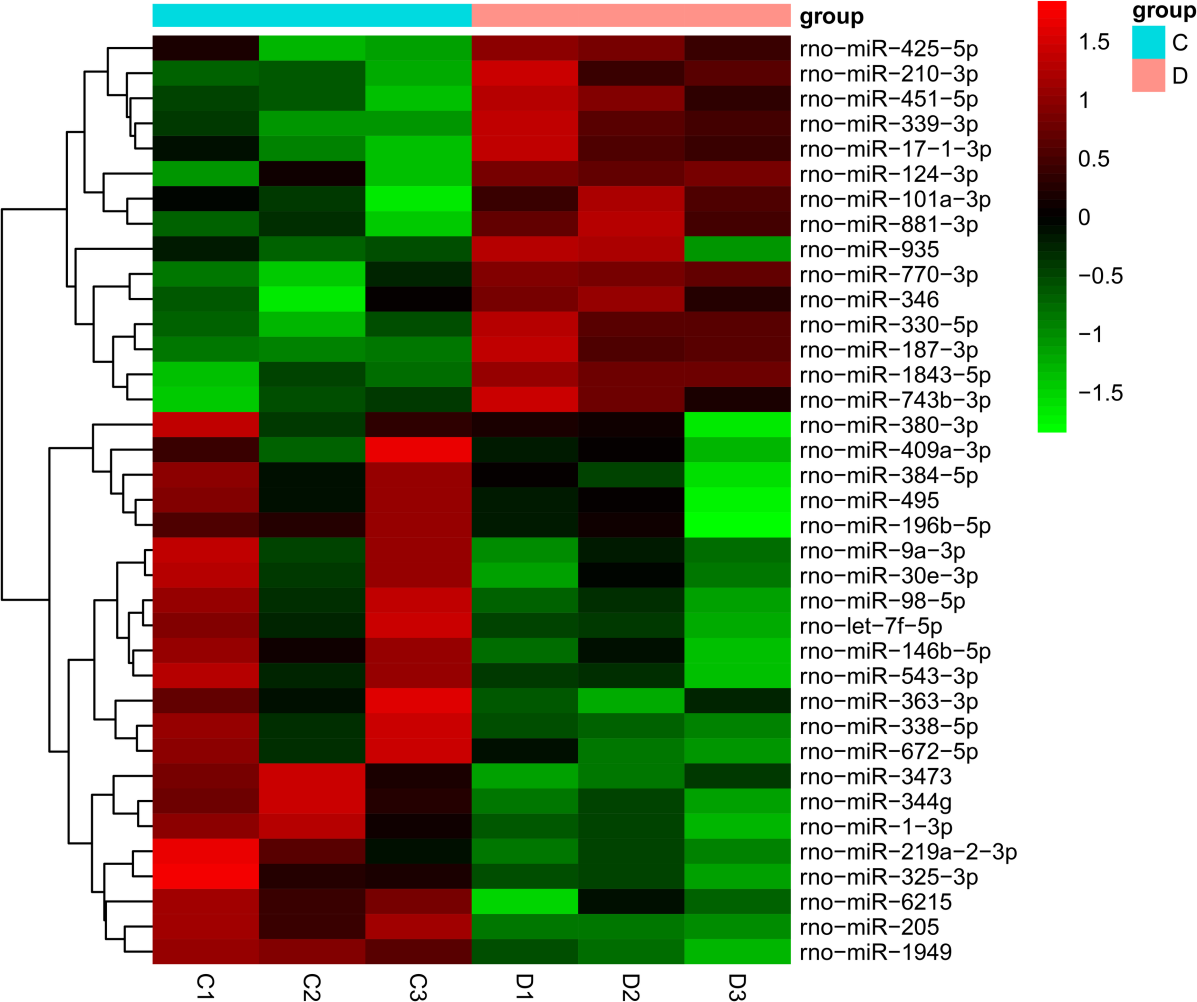
The heat map of aberrantly expressed miRNAs in diabetic rats. A total of 37 significantly deregulated miRNAs were shown in the heat map, with 15 genes up-regulated and 22 down-regulated (n = 3 for diabetic rats and n = 3 for control rats). Red signals and green signals represent upregulated expression and downregulated expression, respectively. C: control rats; D: diabetic rats.

Through microarray-based miRNA expression analysis, 37 miRNAs were found to be significantly differentially expressed in the diabetic group (*P*<0.05). Specifically, 15 and 22 miRNAs were upregulated or downregulated, respectively (S2 Table). The expression levels of these 37 differentially expressed miRNAs are presented by the hierarchical cluster heat map in Fig 2. The mRNA microarray analysis indicated that the expression of 1357 mRNAs was markedly altered in diabetic rats (*P*<0.05). In particular, 616 mRNAs were upregulated and 741 mRNAs were downregulated (S3 Table).

### miRNA target gene prediction and selection

By using the miRanda database, the target mRNAs of miRNAs were predicted. We found 10011 predicted target genes (S4 Table) from the 37 dysregulated miRNAs that we identified. The selected target genes, as described above, were obtained containing 277 target genes (S5 Table), and were used for following analyses.

### GO and pathway analysis

**Fig 3:**
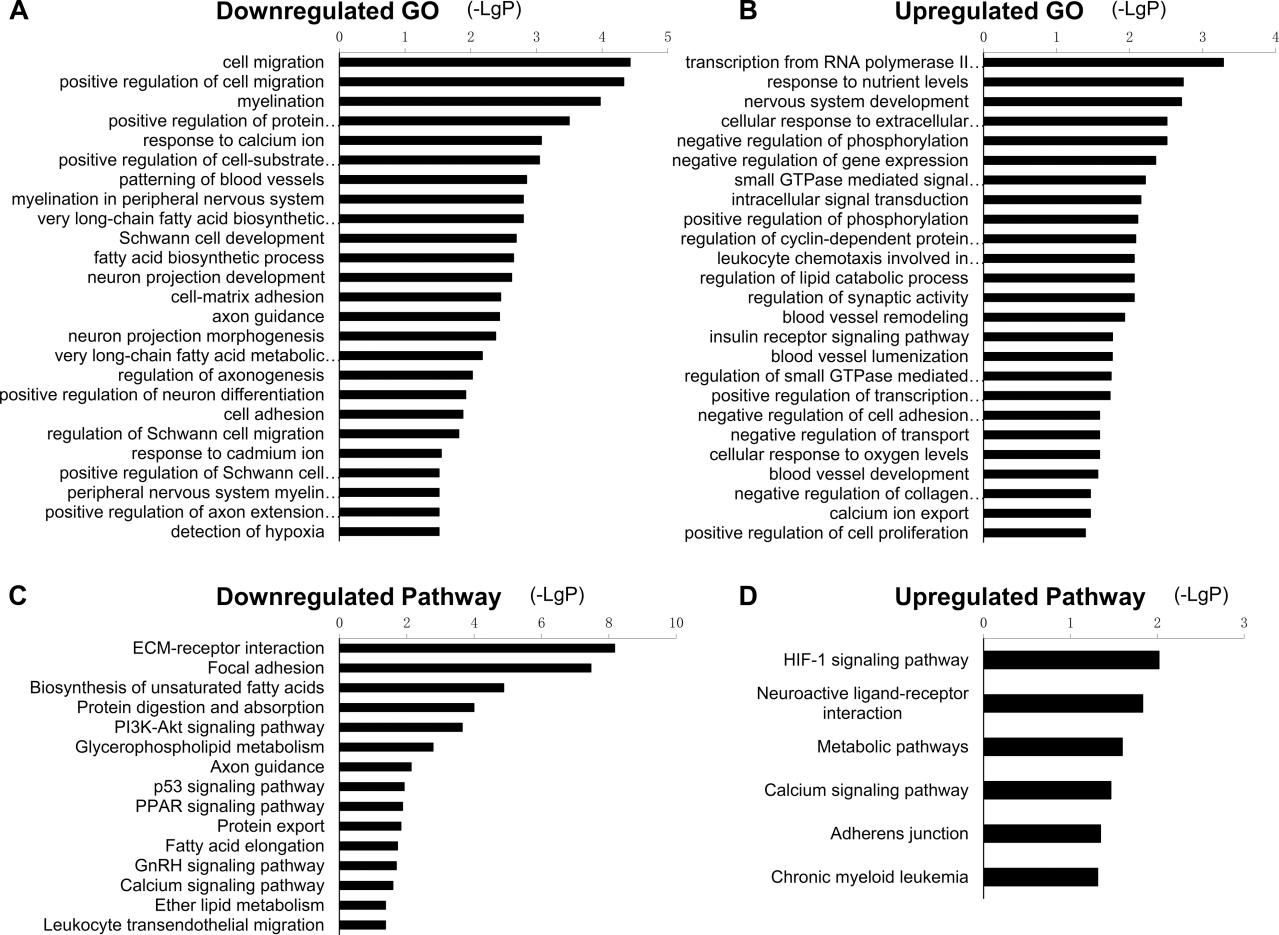
Significant GO terms and pathways of the selected target genes. (A) GO terms of downregulated target genes. (B) GO terms of upregulated target genes. (C) Pathways of downregulated target genes. (D) Pathways of upregulated target genes. The smaller the *P*-value, the larger the -LgP.

GO and pathway analysis were used to identify significant functions and signaling pathways involving the selected target genes. A total of 399 GO terms were enriched (*P*<0.05). Specifically, 142 GO terms were upregulated and 257 GO terms were downregulated. A part of down- and upregulated GO terms are shown in Fig 3A and 3B, and full details are shown in S6 Table. As shown in Fig 3A and 3B, three types of the significantly enriched GO terms attracted our special attention. First, the GO terms related to the abnormalities of myelin and axon; second, those involved in blood vessel changes; third, the GO terms associated with the changes of neurons and Schwann cells. These particular GO terms may be important in the development of DPN. According to KEGG pathway analysis, there were 23 downregulated and 6 upregulated enriched pathways (*P*<0.05) (S7 Table). A fraction of these pathways are presented in Fig 3C and 3D. The top downregulated pathways included the ECM-receptor interaction, focal adhesion, and biosynthesis of unsaturated fatty acids, while the top upregulated pathways included HIF-1 signaling pathway, neuroactive ligand-receptor interaction, and metabolic pathways.

### miRNA-gene-network analysis

**Fig 4:**
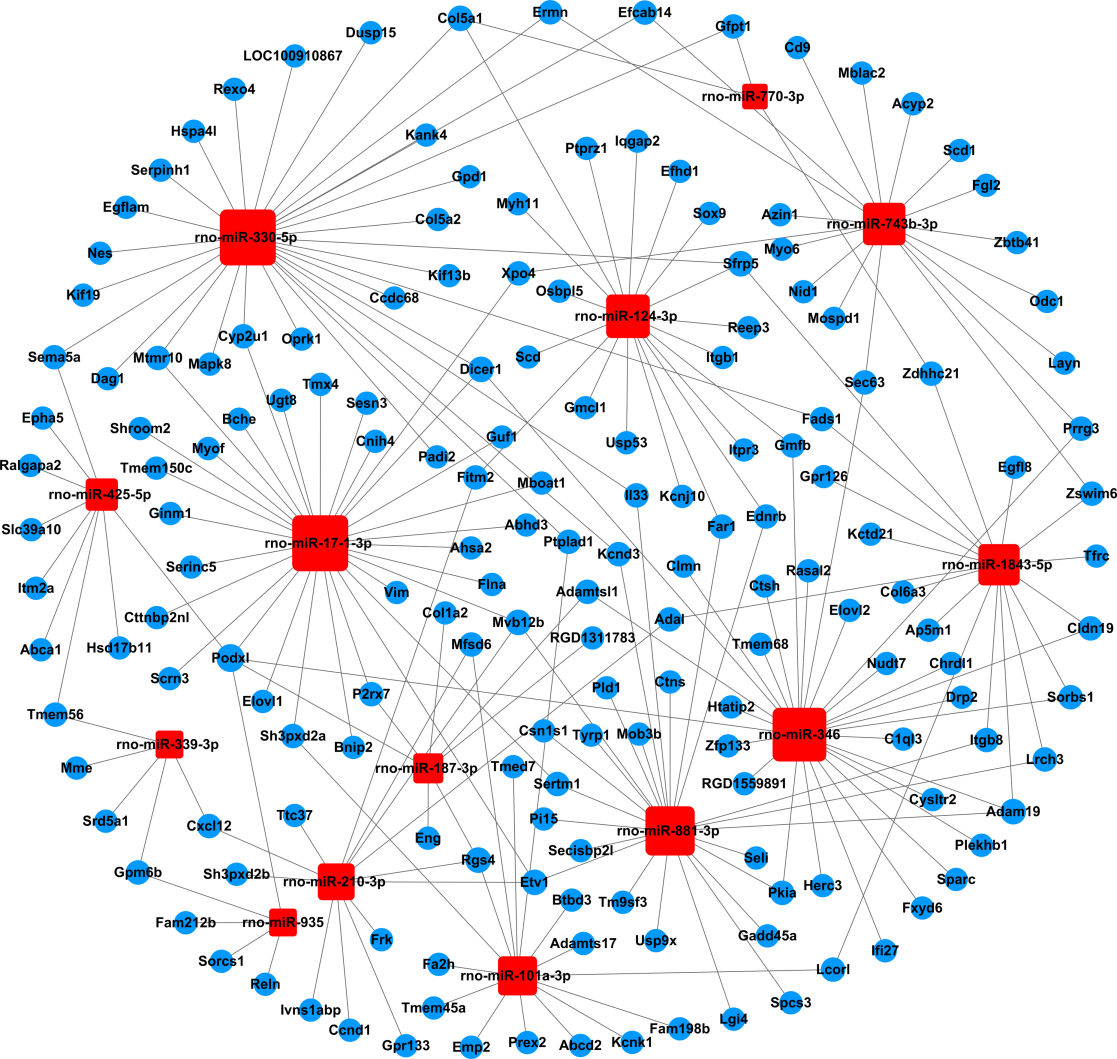
The miRNA-gene-network based on the upregulated miRNAs and their target mRNAs. Square nodes and circular nodes represent miRNAs and mRNAs, respectively. Red color and blue color indicate upregulation and downregulation, respectively. A larger size of a node indicates a higher degree of connectivity.

**Fig 5:**
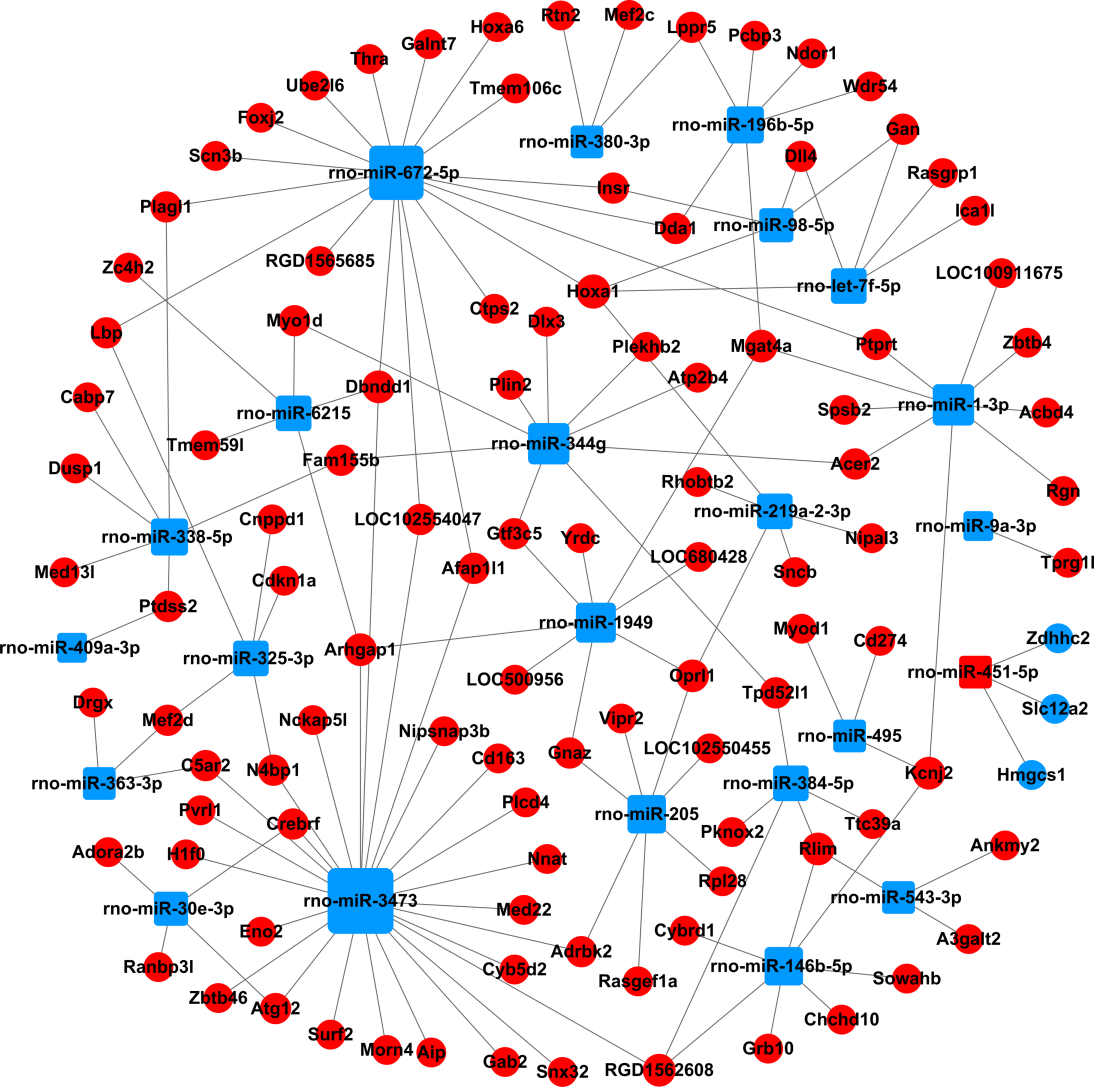
The miRNA-gene-network based on the downregulated miRNAs and their target mRNAs. Square nodes and circular nodes represent miRNAs and mRNAs, respectively. Red color and blue color indicate upregulation and downregulation, respectively. A larger size of a node indicates a higher degree of connectivity.

We built a miRNA-mRNA-network combining the differentially expressed miRNAs and the selected target genes, to identify the key miRNAs and mRNAs in the regulatory network (Figs 4 and 5, S8 Table). A total of 37 miRNAs and 277 mRNAs were included in the network. The degree of connectivity was used to discover the key miRNAs and mRNAs in the regulatory network. By analysis of the network, we found rno-miR-330-5p, rno-miR-17-1-3p and rno-miR-346 had a high degree of connectivity. Meanwhile, podocalyxin-like (Podxl, inhibiting cell-cell adhesion) and homeo box A1 (Hoxa1, sequence specific transcription factor) were the most common target mRNAs, and had the highest degrees of connectivity, 5 and 4, respectively.

### qRT-PCR validation

**Fig 6:**
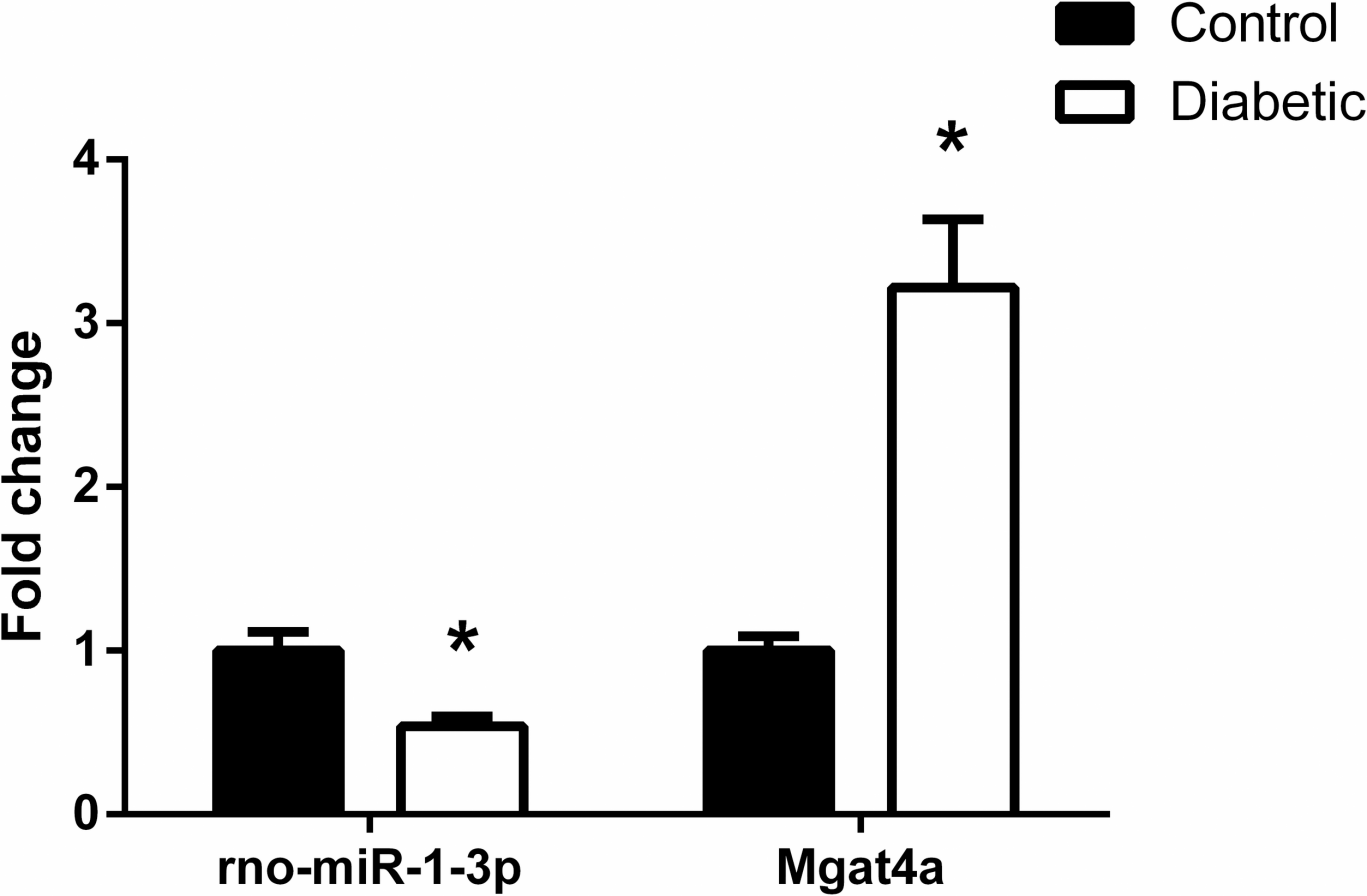
qRT-PCR analysis to validate the microarray results. Consistent with the microarray results, rno-miR-1-3p was downregulated and target gene Mgat4a was upregulated in diabetic group compared to control group. Results were presented as mean±SE of three independent experiments (*P<0.05). Samples used for qRT-PCR analysis were independent of microarray samples.

To test our microarray results, qRT-PCR analysis was performed to determine the expression of rno-miR-1-3p and one of its target gene Mgat4a. Compared with control rats, the expression of rno-miR-1-3p was downregulated and target gene Mgat4a was upregulated in diabetic animals (Fig 6), and the trend was in line with the microarray results.

## Discussion

In the present study, we identified the differentially expressed miRNAs and mRNAs, which may be involved in the development of DPN. Previous investigations were limited on the miRNA expression changes and their relationship with DPN. Cheng et al. have examined the mRNA and miRNA profile alterations in STZ-induced diabetic CD1 mouse model of neuropathy [20], Gong et al. have identified the altered miRNAs in the lumbar spinal dorsal horn from Balb/C mice with diabetic neuropathic pain [21], but both lacking systematic bioinformatics analyses. Here, we focused on the bioinformatics methods to analyze the potential role of aberrantly expressed mRNA and miRNAs in DRGs from STZ-induced diabetic SD rats, to improve our understanding on the relationship between miRNAs and DPN.

Our study applied microarray technology to analyze the differentially expressed genes. Actually, both the microarray technology and the RNA sequencing (RNA-seq) technology are widely used in gene expression research. Compared to microarray, RNA-seq has the advantage to discover novel transcripts, such as novel isoforms and promoters and allele-specific expression. However, the RNA-seq resulting datasets are larger and more complicated, and the interpretation can be challenging [22]. Considering most known lncRNAs had not been studied in DPN, we selected microarray technology to perform our study.

The required sample size for microarray analysis and the success rate of DPN induction were used to estimate the required sample size for our study. As a result, 20 SD rats (10 rats per group) were used. We successfully induced diabetic rats according to the high nonfasting blood glucose levels, polyphagia, polydipsia, polyuria and body weight loss. The minimum duration of diabetes of STZ-injected rats to induce neuropathy can be as early as 2–4 weeks [23]. Consistent with previous studies [24, 25], we chose 8 weeks as our diabetic duration. Also, the paw withdrawal thresholds were significantly reduced in diabetic rats. Taken together, we thoroughly demonstrate that the diabetic rats had developed DPN.

Through GO analysis, we detected 399 GO terms that were significantly enriched. Specifically, the downregulated GO terms including myelination (GO:0042552), myelination in peripheral nervous system (GO:0022011), axon guidance (GO:0007411), regulation of axonogenesis (GO:0050770) were enriched. Interestingly, numerous studies have reported the morphological abnormalities in the myelinated nerve fibers of STZ-induced diabetic rats, such as demyelination, myelin splitting, and axonal atrophy or swelling [26–28].

It is believed that the vascular changes, driven by complex metabolic changes, often lead to the endoneurial hypoxia and blood flow impairment. This could contribute to the pathogenesis of diabetic neuropathy [29]. In agreement with this, our analysis indicated that the upregulated GO terms response to blood vessel remodeling (GO:0001974), blood vessel lumenization (GO:0072554), blood vessel development (GO:0001568), and the downregulated GO terms response to patterning of blood vessels (GO:0001569) and detection of hypoxia (GO:0070483) were enriched.

Additionally, several GO terms related to neurons and Schwann cells were significantly decreased in diabetic rats, for instance, neuron projection development (GO:0031175), neuron projection morphogenesis (GO:0048812), positive regulation of neuron differentiation (GO:0045666), Schwann cell development (GO:0014044), regulation of Schwann cell migration (GO:1900147), and positive regulation of Schwann cell differentiation (GO:0014040). Previous studies have shown that the sensory neurons and Schwann cells in peripheral nervous system are vulnerable to metabolic changes, such as hyperglycemia, dyslipidemia, the oxidative and inflammatory stress. The cell injury caused by these multiple metabolic imbalances eventually leads to the development of diabetic neuropathy [30].

By using the KEGG database, we detected 29 enriched signaling pathways including the HIF-1 signaling pathway, metabolic pathways, calcium signaling pathway, PI3K-Akt signaling pathway, as well as p53 signaling pathway. Both hyperglycemia and hypoxia are two main phenomena of diabetes. Together with common metabolic pathway alterations this could result in complications such as diabetic neuropathy [31]. The master transcription factor hypoxia-inducible factor-1 alpha (HIF-1α) is an important regulator of the expression of multiple target genes, such as vascular endothelial growth factor (VEGF) and erythropoietin. HIF-1α protects tissues from ischemia and infarction, and abnormal response of HIF-1α may be responsible for the changes of diabetic nerve function [32]. Moreover, the tumor-suppressor protein and transcriptional activator p53, is also associated with diabetes and hypoxia, Jazayeri et al. reported that the diabetic ischemia-induced apoptosis is increased through a p53-mediated mechanism [33]. In addition, aberrant calcium (Ca^2+^) homeostasis and signaling in sensory neurons could potentially affect numerous aspects such as primary afferent conduction, neurotransmitter release, and signal transduction, leading to painful and degenerative diabetic neuropathy [34, 35]. The PI3K-Akt signaling pathway is a critical pathway to mediate the survival actions of trophic factors on neuronal cells [36]; hyperglycemia-induced neuronal loss increases through a reduction of PI3K-Akt signaling [37]. In summary, all the above-mentioned pathways may be associated with DPN.

In order to better explore the relationship between the differentially expressed miRNAs and target genes, we built the miRNA-gene-network and found rno-miR-330-5p, rno-miR-17-1-3p and rno-miR-346 to be highly connected, indicating that these miRNAs may play critical roles in the regulatory network. Previous studies have reported that miR-330-5p may be involved in the synaptic loss of hippocampal neuron during Alzheimer's disease pathogenesis [38]; miR-346 negatively regulates Smad3/4 expression by binding to its 3'-UTR, ameliorating the kidney function and histology in diabetic nephropathy [39]. In addition, rno-miR-1-3p, rno-miR-9a-3p, rno-miR-205, rno-miR-363-3p, rno-miR-495, and rno-miR-451-5p have been reported to be involved in diabetic complications. Increased expression of miR-1-3p may be related to long-term diabetes-induced muscle sarcopenia [40]; miR-9a-3p was linked to the impairment of the K_ATP_ channel function and contribute to diabetic vascular complications [41]; miR-205 belongs to oxidative stress related miRNAs, modulates oxidative and endoplasmic reticulum stresses, and may be important to regulate diabetic nephropathy [42]; also, miR-363-3p, miR-495 and miR-451-5p may be used as early biomarkers in patients or rats with diabetic nephropathy [43, 44]. To our knowledge, few studies have reported the relationship between these miRNAs and DPN. Regarding the target genes in the regulatory network, the downregulated gene Podxl was the most highly regulated gene. Larrucea et al. have reported that Podxl enhances cell adherence, cell migration and cellular interaction in an integrin-dependent manner [45]. Interestingly, previous research suggest that decreased expression and improper localization of the adhesion-related molecules in the myelin sheath of diabetic rats, might lead to the reduction of the motor nerve conduction velocity [46]. However, the linkage between Podxl and DPN is still unknown.

In conclusion, the present study successfully identified differentially expressed miRNAs and their target mRNAs in DRGs from STZ-induced diabetic rats. We identified 399 GO terms and 29 pathways, many of them were involved in DPN according to previous studies [26–37], indicating that these genes may be important in the development of DPN. However, it is worth noting that "guilt by association" is usually not the case in real biological systems [47] and we only predicted the functions of dysregulated genes using bioinformatics analyses. In addition, the differentially expressed genes identified in rat DRG tissues may not participate in the same processes in humans. Therefore, future studies should focus on validating the function and related molecular mechanisms of these genes on the pathophysiology of DPN, and their significance to human DPN.

## Supporting information

S1 Checklist Completed “The ARRIVE Guidelines Checklist” for reporting animal data in this manuscript. (PDF)

S1 Table Primer catalog for qPCR analysis. Specific primers of rno-miR-1-3p and target gene Mgat4a for qPCR analysis.
(XLSX)

S2 Table Differentially expressed miRNAs from miRNA microarray experiment results. Information on the upregulated and downregulated miRNAs in diabetic rats.
(XLSX)

S3 Table Differentially expressed mRNAs from mRNA microarray experiment results. Information on the upregulated and downregulated mRNAs in diabetic rats.
(XLSX)

S4 Table Target gene prediction of 37 miRNAs. The list of predicted target mRNAs of 37 differentially expressed miRNAs using the miRanda database.
(XLSX)

S5 Table The selected target genes. The list of the selected target genes.
(XLSX)

S6 Table GO analysis results. Information on upregulated and downregulated GO terms.
(XLSX)

S7 Table Pathway analysis results. Information on upregulated and downregulated pathways.
(XLSX)

S8 Table Information on genes in miRNA-gene-network. The relationships and properties of 37 miRNAs and 277 target mRNAs involved in the network.
(XLSX)
